# Comparing a biosimilar follitropin alfa (Cinnal-fⓇ) with Gonal-fⓇ in women undergoing ovarian stimulation: An RCT

**DOI:** 10.18502/ijrm.v19i11.9917

**Published:** 2021-12-13

**Authors:** Batool Hossein Rashidi, Khashayar Sayyari, Ramin Heshmat, Saeid Amanpour, Ensieh Shahrokh Tehraninejad, Masoumeh Masoumi, Farhang Rezaei

**Affiliations:** ^1^Vali-e-Asr Reproductive Health Research Centre, Tehran University of Medical Sciences, Tehran, Iran.; ^2^Faculty of Pharmacy, Tehran University of Medical Sciences, Tehran, Iran.; ^3^Chronic Diseases Research Center, Endocrinology and Metabolism Population Sciences Institute, Tehran University of Medical Sciences, Tehran, Iran.; ^4^Medical Department, Orchid Pharmed Company, Tehran, Iran.

**Keywords:** Follitropin alfa, Biosimilar, Efficacy, Safety, Intracytoplasmic sperm injection.

## Abstract

**Background:**

Advances in recombinant DNA technology led to the development of recombinant follitropin alfa. Recombinant human follicle-stimulating hormone products are used to stimulate follicular maturation.

**Objective:**

To compare the efficacy and safety of a biosimilar-candidate recombinant human follicle-stimulating hormone (Cinnal-fⓇ; CinnaGen, Iran) with the reference product (Gonal-fⓇ; Merck Serono, Germany) in women undergoing ovarian stimulation for intracytoplasmic sperm injection (ICSI).

**Materials and Methods:**

In this randomized controlled trial, a total sample size of 200 women (age < 35 yr, candidate for ICSI) was calculated. Participants began a pituitary downregulation protocol with buserelin. They received 150 IU daily of either Cinnal-fⓇ or Gonal-fⓇ from the second day of their cycle. The primary outcome of the study was the percentage of metaphase II (MII) oocytes. The secondary outcomes included the number and quality of oocytes retrieved, duration of stimulation, fertilization rate, embryo quality, the number of clinical and ongoing pregnancies, and the incidence of ovarian hyperstimulation syndrome (as an important safety marker).

**Results:**

A total of 208 women were enrolled, of whom, 200 completed the study period. Ovarian stimulation with Cinnal-fⓇ resulted in a comparable percentage of MII oocytes as with Gonal-fⓇ (78.64% vs 80.02%, respectively; p = 0.81). No statistically significant difference was seen in the secondary outcomes between the groups.

**Conclusion:**

Cinnal-fⓇ proved non-inferior to Gonal-fⓇ, based on the percentage of MII oocytes in women aged < 35 yr undergoing ICSI. Our findings confirm that the efficacy and safety profiles of Cinnal-fⓇ and Gonal-fⓇ are similar.

## 1. Introduction

Infertility is a crucial component of reproductive health that has a considerable physical, psychological and financial burden on those who are affected by it (1). It has been estimated that the prevalence of infertility in women who are of reproductive age is about 14% of couples in developed countries and 25% in developing countries. In some areas of the world, including the Middle East and North Africa, infertility rates may reach 30% (2). Recently, a meta-analysis by Parsanezhad reported a 10.9% prevalence rate of lifetime infertility in Iran (3).

In vitro fertilization (IVF) of oocytes in humans followed by embryo implantation in the uterine cavity is a common therapy for infertility in some cases. However, in severe male-factor infertility, intracytoplasmic sperm injection (ICSI) that requires a single sperm to be directly injected into a mature oocyte yields better outcomes (4).

Several studies have used a long pituitary downregulation protocol followed by ovarian stimulation with a daily subcutaneous injection of 150 international units (IU) recombinant human follicle-stimulating hormone (r-hFSH) for their IVF/ICSI procedures (5, 6). A meta-analysis by Matorras and colleagues showed that intrauterine insemination follicular stimulation with r-hFSH resulted in a higher pregnancy rate per cycle than when highly purified urinary follicle-stimulating hormone (FSH) was used at equal dose. In addition, pregnancy rates were similar when the dose of r-hFSH was 50% lower than the highly purified urinary FSH dose (7).

FSH products are used to stimulate the growth and maturation of multiple follicles, develop several embryos, and thus improve the probability of having a live birth (8). In the past, all available FSH preparations were obtained from postmenopausal women's urine extracts. These products had some drawbacks including batch-to-batch inconsistency, low specific activity, and the presence of impurities (9). Nevertheless, advances in recombinant DNA technology led to the development of highly purified, highly specific recombinant human FSH. In September 1997, follitropin alfa Gonal-fⓇ (Merck Serono, Germany) received the Food and Drug Administration approval for ovulation induction and use in conjunction with assisted reproductive technologies, such as IVF (10).

Due to the elaborate manufacturing process of recombinant biological products, they are often high-priced. This fact may limit individuals' access to high-quality infertility treatments. Through verification of similar physicochemical and biochemical properties in non-clinical studies and by demonstrating comparable efficacy and safety as the reference product, biosimilars can enhance access to high-quality biological products. Cinnal-fⓇ (CinnaGen, Iran) was developed in genetically modified Chinese hamster ovary cells as a biosimilar to the innovator Gonal-fⓇ and received the approval of the Food and Drug Administration of Iran in June 2013 (IRC: 3125387962296341).

The aim of the present study was to compare the efficacy and safety of Cinnal-fⓇ with Gonal-fⓇ in women undergoing ICSI.

## 2. Materials and Methods 

This study was a prospective, triple-blind, randomized controlled trial with a total sample size of 200 participants, which was conducted on women who referred to the Vali-e-Asr Reproductive Health Research Center (Imam Khomeini Hospital, Tehran, Iran), a tertiary university-based infertility center between April 2011 and April 2013.

Women indicated for ICSI were included in the study if they met the following criteria: age < 35 yr; FSH levels ≤ 8 mIU/mL on the second day of the cycle; regular menstrual cycles of 25-35 days; and receiving gonadotropin-releasing hormone (GnRH) agonist for pituitary suppression. Participants were excluded from the study if they: had a history of previous ovarian surgery or hyperstimulation syndrome; had any endocrinopathy or an abnormal hormonal profile; smoked cigarettes or were allergic to Gonal-fⓇ Cinnal-fⓇ or any of their components.

Starting on day 21 of the previous cycle, all eligible participants received a 0.6-mg daily subcutaneous dose of the GnRH agonist, buserelin (SuprefactⓇ; Hoechst, Germany). An ultrasound scan was performed to confirm pituitary downregulation by showing no evidence of ovarian activity. Cases were then randomized into one of the two treatment groups (1:1 allocation ratio by permuted block randomization) – Cinnal-fⓇ (follitropin alfa; CinnaGen; Iran), or Gonal-fⓇ (follitropin alfa; Merck Serono; Germany) – and treated based on their identification numbers. Both groups were given 150 IU/day of r-hFSH subcutaneously for five days commencing on day two of the cycle. On day seven or eight of the cycle, an ultrasound scan was used to assess the ovarian response, and the r-hFSH dosage was adjusted according to the result. Buserelin and r-hFSH were given until the final follicular maturation requirements (one follicle with a diameter of ≥ 18 mm and at least three follicles with a diameter of ≥ 14 mm) were fulfilled. Human chorionic gonadotropin (HCG; Merck Serono; Germany) was given (10000 IU, intramuscularly) 24 hr after the administration of the last r-hFSH dose for triggering ovulation. Oocytes were retrieved 36 hr after the HCG administration; two hr later, cumulus oophorus cells were removed from the oocytes using hyaluronidase enzyme. The oocytes were then assessed for their nuclear maturity (metaphase I, metaphase II [MII], germinal vesicle, and degenerated oocyte), and the appearance of their zona pellucida, ooplasm, and polar bodies. The zona pellucida was considered abnormal if it was dark-colored, fractured, or thicker than normal. Uniform and non-granular ooplasms and intact, round, or ovoid polar bodies were regarded as normal. We performed ICSI on MII oocytes and evaluated oocytes' fertilization 24 hr later. The appearance of two pronuclei was the sign of successful oocyte fertilization; however, if no or only one pronucleus was available, the assessment was repeated 3–4 hr later. Embryos were then transferred to the uterus, at most two-three days after the fertilization, using a Labotect embryo replacement catheter (Labotect GmbH, Gottingen, Germany). Other good-quality embryos were frozen.

The luteal phase was supported by 400 mg/day progesterone (CyclogestⓇ; Actavis, Ireland) administered vaginally, starting from the day after the ovum pick-up until a negative pregnancy test was obtained. In case the pregnancy was approved by ultrasonography, progesterone administration was continued for at least eight wk of pregnancy.

Both Cinnal-fⓇ and Gonal-fⓇ vials were relabeled and coded by an independent party to become indiscernible from each other. All participants, clinicians, embryologists, and statistical assessors were blinded to the allocated treatments. However, a person who was not blinded had the randomization list and was aware of the dispensed medicines. The only intervention of this person was to inform clinicians by phone about which set of coded vials they should administer for the cases.

The primary outcome was the percentage of MII oocytes in women undergoing ICSI, assessed on the oocyte retrieval day by an embryologist. Physicians and embryologists assessed secondary outcomes, which are described in the following. The number of oocytes retrieved from participants who received HCG, quality of retrieved oocytes, cytoplasm morphology, and polar body shape were evaluated simultaneously with the primary outcome. Also, the number of FSH vials used during stimulation and the duration of FSH stimulation were measured at the end of ovarian stimulation. The oocytes' fertilization rate and the number of two-pronuclear fertilized oocytes were assessed 24 hr after oocyte retrieval. In addition, embryo quality and the number of embryos replaced or frozen were determined 2-3 days after oocyte fertilization. Finally, the number of clinical and ongoing pregnancies were counted after 5-8 wk of gestation and after ≥ 20 completed wk of gestation, respectively.

The incidence of ovarian hyperstimulation syndrome (OHSS) was also recorded as an important safety marker throughout the study.

### Ethical considerations

The study was authorized by the research ethics committee of Tehran University of Medical Sciences (Code: 9957) as the institutional review board and was approved by Iran's Ministry of Health and Medical Education. The trial is registered on the Iranian Registry of Clinical Trials. We followed the principles of the Declaration of Helsinki and Good Clinical Practice Guidelines of the International Conference on Harmonization. All individuals provided a written informed consent to participate in the study.

### Statistical analysis

The non-inferiority of Cinnal-fⓇ and Gonal-fⓇ would be declared if the difference in the percentage of MII oocytes (the primary outcome of the study) between the two groups hit an alpha error of 2.5%. A sample size of 200 participants was calculated with the hypothesis of H ${}_{0}$ : P ${}_{\mathrm{Gonal}-f}$ - P ${}_{\mathrm{Cinnal}-f}$ ≥ 0.16 and H ${}_{1}$ : P ${}_{\mathrm{Gonal}-f}$ - P ${}_{\mathrm{Cinnal}-f}$ < 0.16. The assumed maximum allowable difference was estimated based on 20% of effect size reported for the comparator group. All computations were performed using Stata version 11 (Stata Corp, College Station, USA) software. The two treatment groups were compared using the Mann-Whitney U test and two independent *t* tests for continuous variables, and Chi-square and Fisher's exact test for categorical variables. The incidence of adverse events in the two groups was compared using Fisher's exact test. P *<* 0.05 was considered as statistically significant. Data are presented as mean ± standard deviation (SD).

## 3. Results

**Figure 1:**
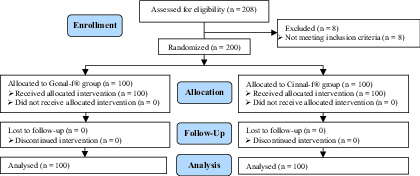
Participants' disposition scheme.

A total of 208 women were enrolled in this trial, of which, 8 cases were reported as screening failures (previous history of OHSS). A total of 200 participants were randomized to receive either Cinnal-fⓇ (n = 100) or Gonal-fⓇ (n = 100). Interestingly, all women completed the treatment cycle after randomization, and no one was excluded for any personal or clinical reasons (Figure 1).

Table I summarizes the baseline characteristics of the participants. Cases that were candidates for ICSI had various conditions, including previous unsuccessful fertilization, moderate-to-severe male infertility, egg fertilization abnormalities, and unexplained infertility.

### Efficacy results

**Figure 2:**
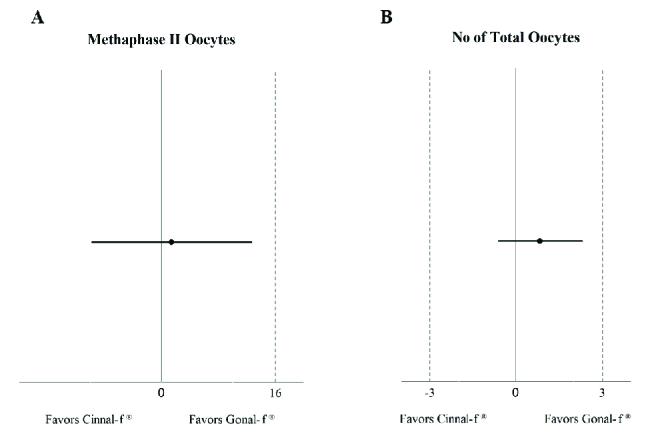
Forrest plot for the difference between Gonal-fⓇ and Cinnal-fⓇ in terms of (A) The proportion of MII oocytes, Difference (95% CI): 1.4 (-9.8, 12.6) and (B) The total number of oocytes, Difference (95% CI): 0.83 (-0.6, 2.3).

The rate of oocytes in the MII stage of nuclear development was 80.02% and 78.64% for the Gonal-fⓇ and Cinnal-fⓇ groups, respectively. Since the upper limit of the 95% confidence interval (CI) of difference was lower than the predetermined non-inferiority margin, Cinnal-fⓇ proved non-inferior to Gonal-fⓇ in terms of the proportion of MII oocytes (Figure 2A).

There was no significant difference between the Gonal-fⓇ and Cinnal-fⓇ groups in terms of the total number of oocytes (95% CI-0.6, 2.3). The difference in the percentage of germinal vesicles and metaphase I oocytes was not statistically significant between the treatment groups. Both groups had comparable follicular characteristics in terms of the polar body, zona pellucida, and ooplasm (Table II). The median ± interquartile range (IQR) of the number of transferred embryos was 2 ± 0 in both the Cinnal-fⓇ and Gonal-fⓇ groups. The mean number of frozen embryos was 3.14 ± 3.33 and 2.33 ± 2.66 in the Cinnal-fⓇ and Gonal-fⓇ groups, respectively.

The mean ovarian stimulation duration was 9.9 ± 2.1 days in the Gonal-fⓇ group and 9.6 ± 1.8 days in the Cinnal-fⓇ group (p = 0.39). No difference in the total number of vials used was observed (p* =* 0.47), as 20.6 ± 4.9 and 20.1 ± 4.5 vials were used in the Gonal-fⓇ and Cinnal-fⓇ groups, respectively. Although there was a trend toward higher rates of ongoing and clinical pregnancy in the Gonal-fⓇ group, the difference compared with the Cinnal-fⓇ group was not statistically significant.

There was also no statistically significant difference in participants' clinical and ongoing pregnancy rates (Table III), the percentage of oocytes developing two pronuclei (Cinnal-fⓇ 71% vs. Gonal-fⓇ 74.8%, p = 0.55), or in the number of cryopreserved embryos (p = 0.55).

### Safety results

The adverse events in participants taking Cinnal-fⓇ were relatively similar to those observed in women receiving Gonal-fⓇ. Four cases of OHSS were reported in the Cinnal-fⓇ group and seven cases in the Gonal-fⓇ group (p = 0.53). None of these cases was serious and/or required hospitalization because of the OHSS.

**Table 1:** Baseline characteristics of the study participants (n = 100/each)


**Characteristic**	**Gonal-fⓇ group**	**Cinnal-fⓇ group**
Age (yr)*	29.2 ± 3.8	28.6 ± 3.9
Infertility duration (yr)*	6.2 ± 4.0	5.5 ± 3.6
BMI (kg/m ${}^{2}$ )*	23.6 ± 2.5	23.6 ± 2.0
FSH (mIU/mL)*	5.1 ± 1.9	5.1 ± 1.8
LH (IU/L)*	4.1 ± 2.2	4.0 ± 1.9
AMH (ng/mL)*	3.2 ± 1.6	2.9 ± 1.5
Prolactin (ng/mL)**	10.2 (25.8)	13.9 (15.1)
*Data presented as Mean ± SD, **Data presented as median (IQR). BMI: Body mass index, FSH: Follicle stimulating hormone, LH: Luteinizing hormone, AMH: Anti-Müllerian hormone, SD: Standard deviation, IQR: Interquartile range		

**Table 2:** Cycle and oocyte characteristics


** Characteristic**			**Gonal-fⓇ group (n = 100)**	**Cinnal-fⓇ group (n = 100)**	**Treatment difference**	**p-value**
Total number of oocytes*			9.96 ± 5.7	9.13 ± 4.5	0.83 (–0.6, 2.3)	0.26 ${}^{‡}$
Nuclear maturity**						
	**Germinal vesicles**		80 (8.03)	83 (9.09)	0.79 ${}^{††}$
	**Metaphase I oocytes**		81 (8.13)	61 (6.68)	0.70 ${}^{††}$
	**Metaphase II oocytes **		797 (80.02)	718 (78.64)	1.4 (–9.8, 12.6)	0.81 ${}^{††}$
** Follicular status**						
	Polar body***					
	**Normal **	7 (6)	7 (5)	0.68 ${}^{†}$
	**Abnormal**	0.5 (2)	0 (2)	0.80 ${}^{†}$
	Zona pellucida***					
	**Normal**	7 (6)	7 (5)	0.79 ${}^{†}$
	**Abnormal**	0 (2)	0 (2)	0.80 ${}^{†}$
	Ooplasm***					
	**Normal**	6 (6)	7 (5)	0.88 ${}^{†}$
	**Abnormal**	0 (2)	0 (3)	0.85 ${}^{†}$
Right ovary follicles*			6.4 ± 2.9	6.2 ± 3.0	0.52 ${}^{‡}$
Left ovary follicles*			5.9 ± 3.2	5.8 ± 2.8	0.83 ${}^{‡}$
Endometrium thickness*			9.0 ± 1.7	9.2 ± 1.9	0.50 ${}^{‡}$
*Data presented as Mean ± SD, **Data presented as n (%), ***Data presented as median (IQR). Data were compared by ${}^{‡}$ Two sample independent *t* test, ${}^{†}$ Mann–Whitney U test, ${}^{††}$ Chi-square test. SD: Standard deviation, IQR: Interquartile range						

**Table 3:** Ovarian stimulation status and pregnancy outcomes


**Variable**	**Gonal-fⓇ group (n = 100)**	**Cinnal-fⓇ group (n = 100)**	**p-value**
Ovarian stimulation duration (days)*	9.9 ± 2.1	9.6 ± 1.8	0.39 ${}^{‡}$
Number of used vials*	20.6 ± 4.9	20.1 ± 4.5	0.47 ${}^{‡}$
β -HCG positive**	40 (40.4)	30 (30.6)	0.15 ${}^{†}$
Clinical pregnancy**	36 (36.4)	27 (27.6)	0.19 ${}^{†}$
Ongoing pregnancy**	31 (32.6)	23 (23.7)	0.17 ${}^{†}$
*Data presented as Mean ± SD, **Data presented as n (%). Data were compared by ${}^{‡}$ Two sample independent *t* test, ${}^{†}$ Chi-square test. β-HCG: Beta human chorionic gonadotropin			

## 4. Discussion

The findings of this randomized, parallel-group trial revealed that Cinnal-fⓇ has similar efficacy and safety profiles as the innovator product in infertile women undergoing ICSI. The population for this trial fulfilled the expected eligibility criteria and was adequately balanced. We chose the percentage of MII oocytes as our primary outcome measure since it was argued that it reflects the quality of stimulation rather than fertilization which depends on several factors, including sperm quality, culture conditions, and the technique and method of the ICSI process (11). According to a study in 2005, 80.6% of oocytes retrieved from 30 cases who underwent ovarian stimulation by r-hFSH were in the MII group (12). The percentage of MII oocytes following the Cinnal-fⓇ treatment was non-inferior to that obtained with Gonal-fⓇ, based on the prespecified non-inferiority margin (Figure 2A).

In 2013, the European Medicines Agency issued a guideline regarding the development of biological medicinal products containing r-hFSH. This guideline recommends that the equivalent efficacy in the number of oocytes retrieved should be considered as a primary outcome for proving biosimilarity of an r-hFSH product (13). Since the present study was conducted before the publication of this guideline, we did not set our primary outcome accordingly. Nevertheless, if we test equivalence with a margin of ± 3 oocytes for the total number of retrieved oocytes, the difference will be 0.83 oocytes (95% CI - 0.6, 2.3) which demonstrates the clinical equivalency in the total number of oocytes (Figure 2B). In 2015 an article was published of a multicenter phase-three study evaluating the efficacy and safety of another biosimilar follitropin alfa in comparison with Gonal-fⓇ in women undergoing ovarian stimulation for IVF. The reported number of oocytes retrieved from 113 women who were treated with Gonal-fⓇ was 10.4 ± 6.14 (14). In a study performed to compare the efficacy and the safety of r-hFSH with urinary FSH for stimulating follicular development in women aged 18-38 yr undergoing IVF, 63 women underwent ovarian stimulation by r-hFSH. These participants were pretreated with buserelin. The mean retrieved oocytes of the r-hFSH group was 9.3 ± 5.0 (15). In 2000 an article was published based on a prospective and randomized study of ovarian stimulation with r-hFSH vs. highly purified urinary FSH in women referred to an ICSI program. Sixty cases aged < 37 yr received a fixed dose of r-hFSH. An average of 10.7 ± 6.8 oocytes were collected from individuals of the r-hFSH group (16). Our results for the total number of retrieved oocytes were in line with those obtained in other studies.

In a clinical trial in 1999 comparing r-hFSH and highly purified urinary FSH in women undergoing ovarian stimulation, 9.9 days was reported as the treatment duration for the r-hFSH group (17). In another phase-three study in 2016, comparing the efficacy and safety of a biosimilar r-hFSH with Gonal-fⓇ, the treatment duration was 9.7 days for the Gonal-fⓇ group (18). Ovarian stimulation duration was comparable between the treatment groups and matched those observed in similar studies. In addition, there was no statistically significant difference in the mean number of vials used or in the clinical and ongoing pregnancy rates. Our findings in this regard were generally in line with previous studies (5, 6, 18).

A seminal study suggested that the number of embryos in the culture at the time of embryo transfer can be an important predictor of pregnancy outcome (8). Our findings indicated that there was no significant difference in the number or quality of embryos between the Cinnal-fⓇ and Gonal-fⓇ groups. In addition, no significant difference was noted in the number of transferred or frozen embryos between the groups.

The safety profile of Cinnal-fⓇ was found to be similar to that of Gonal-fⓇ. All episodes of OHSS were resolved with no need for hospitalization. Careful monitoring of treatment through ultrasonography and a low dose of FSH can minimize the risk of developing OHSS.

Since this trial was aimed to prove non-inferiority of Cinnal-fⓇ compared to the innovator product, it was underpowered to show the significant differences in the adverse effects between the products. This study was conducted in a referral infertility center in Iran. Therefore, we may have underestimated the influence of race on our findings. Another limitation of this study is that it did not evaluate live birth rate. Hence, to take the impact of these factors into account, a more comprehensive post-marketing study is needed.

## 5. Conclusion

Cinnal-fⓇ was non-inferior to Gonal-fⓇ in terms of the proportion of MII oocytes in women younger than 35 yr undergoing ICSI. The results of this study demonstrated that Cinnal-fⓇ is favorably comparable to Gonal-fⓇ in stimulating the development of mature oocytes in preparation for ICSI. The efficacy and safety profile of the reference product and biosimilar-candidate were comparable.

##  Conﬂict of Interest 

Farhang Rezaei is an employee of Orchid Pharmed, which is a CinnaGen partner in conducting clinical trials. This study was funded by CinnaGen Co., Iran. Batool Hossein Rashidi, Khashayar Sayyari, Ramin Heshmat, Saeid Amanpour, Ensieh Shahrokh Tehraninejad, and Masoumeh Masoumi declare that they have no conflict of interest.
